# Musculoskeletal Pain in Family Caregivers: Does a Therapeutic Physical Program in Primary Care Work? A Randomized Controlled Trial

**DOI:** 10.3390/ijerph20010185

**Published:** 2022-12-23

**Authors:** Federico Montero-Cuadrado, Laura Barrero-Santiago, Rocío Llamas-Ramos, Inés Llamas-Ramos

**Affiliations:** 1 Unit for Active Coping Strategies for Pain in Primary Care, East-Valladolid Primary Care Management, Castilla y Leon Public Health System (Sacyl), 47011 Valladolid, Spain; 2 Department of Cell Biology, Genetics, Histology, and Pharmacology, Faculty of Medicine, University of Valladolid, Av. Ramón y Cajal 7, 47005 Valladolid, Spain; 3 Department of Nursing and Physiotherapy, Faculty of Nursing and Physiotherapy, Universidad de Salamanca, Avda. Donantes de Sangre s/n, 37007 Salamanca, Spain; 4 University Hospital of Salamanca, P.º de San Vicente, 182, 37007 Salamanca, Spain

**Keywords:** caregivers, female, primary healthcare, physiotherapy, pain, exercise

## Abstract

Background: Family caregivers play a crucial role in the overall healthcare system and in our society. The elderly population is significantly increasing, which creates a high demand for family caregivers. Few studies have investigated the impact of caregiving on musculoskeletal pain or proposed an active approach for dealing with it. Objectives: To determine and characterize musculoskeletal pain in female family caregivers (FFCs) and assess the effects of adding a therapeutic exercise program to a family caregiver care program (FCCP) on the quality of life, physical conditions, and psychological well-being of FFCs. Methods: A multicenter randomized controlled clinical trial was conducted with 68 FFCs recruited in two public healthcare areas. The intervention and control groups received the same conventional FCCP for 6 h across 4 sessions. The intervention group received an additional 36 sessions of physical therapeutic exercise (PTE) program over 12 weeks. Results: All caregivers reported having pain in particular locations. Lower back pain and neck pain were the locations most frequently cited, with a prevalence of 69.4% and 56.7%, respectively. In total, 80% of participants presented moderate pain intensity. The intervention group showed a significant decrease in the intensity of the pain (*p* < 0.001), as well as in anxiety, depression, subjective burden perception (*p* < 0.01), and quality-of-life variables, including MCS (mental component summary) (*p* < 0.05) and PCS (physical component summary) (*p* < 0.001). Conclusions: A PTE program improved the musculoskeletal pain of FFCs in a clinically relevant way. The caregivers who improved the most were those who initially presented the most intense pain, had the greatest levels of disability, and had the lowest quality of life.

## 1. Introduction

The elderly population is growing in developed countries. Spain is considered one of the countries with the highest levels of aging [1]. It is estimated that those over 65 years of age will account for 26.5% of the population in 2035 [2]. Along with this aging population comes an increasing need for caregivers. Levels of disability have increased and dependency is projected to increase, according to 30-year estimates [1]. In the United States, an increase in care has been reported, rising by 9.5 million caregivers from 2015 to 2020 and with a current total of 53 million caregivers [3]. According to the European Health Survey in Spain in 2020, the number of people with disabilities increased with increasing age; 20.7% of the population aged 65 and over had difficulty in carrying out some of the basic activities of daily living (ADL). In the “85 and over” age group, limitations affected 53.68% of people [4]. In fact, 89.4% of dependent people received informal care, 13% had private care services, and 8.1% received support from public services [5]. Thus, family caregivers, also known as informal caregivers, play a crucial role in the overall healthcare system and in our society.

Informal or family care implies that the caregiver is outside the formal social protection structure and that the care is not bureaucratized [6]. Informal care is carried out by people within the care recipient’s social network and is characterized by the fact that it is not occasional, there is no contractual relationship, and it always involves an affective relationship between the provider and the dependent person, or is at least based on prior knowledge of the other person [6].

The typical caregiver profile is that of a middle-to-low income woman, aged between 45 and 65 years, who is related to, voluntarily assumes responsibility for, and lives in the same household as the person being cared for; she is often a daughter or wife of this person [7,8,9]. Caregiving has an impact on the health of female family caregivers (FFCs). The proportion of female caregivers ranges between 60% and 85% of all caregivers [10]. There continues to be a great gender imbalance in the care of dependent family members. 

Until a few years ago, studies of the health conditions of family caregivers focused exclusively on the characteristics of the dependent person being cared for (degree of dependence, cognitive capacity, etc.). However, in recent years, it has been shown that the health of the family caregiver is more influenced by the caregiver’s own variables than by variables related to the cared-for person (coping skills, self-esteem, subjective burden, perceived social support, etc.) [11]. The literature reflects the significant physical, psychological, and social repercussions suffered by family caregivers, with the consequent loss of quality of life [12,13]. Thus far, to improve this situation, several caregiver care programs have been implemented. These programs include several types of interventions, such as emotional support and self-help groups, respite interventions, cognitive behavioral therapy, mutual support groups, care education programs, and psychological interventions. In Spain, the majority of the family caregiver care program (FCCP) has been developed in primary care (PC). This type of program is also available through the SACYL (public health service of Castilla y León). Although the benefits of this kind of program are clear, there is currently no active approach in which the caregiver is involved. 

It is remarkable that pain and disability are among the most limiting aspects of quality of life. Numerous articles on caregivers allude to the musculoskeletal pain suffered by caregivers; however, the subject is not addressed in sufficient depth [14,15]. In addition, most publications report that the musculoskeletal pain of caregivers has mechanical causes [16,17]. Currently, the literature on this topic mainly focuses on quality of life, but it has been shown that pain is one of the things that most limits quality of life in the general population [18,19]. Blyth demonstrated that caregivers with pain reported more psychological stress and poorer health than caregivers without pain [15]. The benefits and advantages of physical exercise are widely recognized; these benefits are not only physical, but cognitive, emotional, and social [20]. Clinical practice guidelines insist that physical exercise is one of the first lines of treatment for musculoskeletal pain [21,22]. Nevertheless, few studies have evaluated the effects of exercise on caregivers [23,24,25,26,27,28]. 

To date, and to our knowledge, no research has been conducted that examines the capability of physical therapeutic exercise (PTE) programs to improve pain, physical condition, and quality of life in family caregivers of dependent patients in public health systems. 

Therefore, and based on the research carried out in our first study [29], the objectives of this study were to determine and characterize musculoskeletal pain in FFCs, as well as to assess the effects of adding a therapeutic exercise program to an FCCP of the SACYL on the quality of life, physical condition, and psychological well-being of FFCs. Finally, we analyzed correlations between the initial situation of the caregivers and changes in the outcome variables.

## 2. Materials and Methods

A multicenter randomized controlled clinical trial (RCT) was conducted with family caregivers of dependent patients in two basic health areas of the SACYL in Valladolid (Spain) between February and May 2017. The trial was conducted in accordance with the recommendations of the SPIRIT statement [30] and the CONSORT statement [31]. In addition, the PRECIS-2 tool was used for the study design [32]. The study was approved by the Ethical Committee for Clinical Research (CEIC) of Valladolid-East Health Area (CEIC: PI-13-88) and registered in ClinicalTrials.gov (NTC03675217) (accessed on 28 September 2022). 

### 2.1. Participants

The study population corresponded to all of the family caregivers of dependent patients belonging to two medical public centers in the Valladolid-East Health Area of the SACYL, in the Pilarica and Circular centers. The sample was recruited in these two health areas. All family caregivers included in the FCCP program had a consultation with a nursing professional, were informed of the research, and were offered the possibility of participating in the study. Among the caregivers who attended these consultations in the recruitment phase, 4 refused to participate in the study. The caregivers who showed a willingness to participate in the study were referred to members of the research team (doctoral student and/or a nurse) who explained to each caregiver in more detail the objectives and methodology of the research. If the caregiver met the inclusion criteria, they was given the informed consent form, asked to read it carefully, and provide their consent to participate after asking any questions. At the beginning of the project, 127 family caregivers were identified across the two centers. Among these, 68 notified their willingness to participate and met the inclusion criteria, which were: female, over 18 years of age, no changes in medication for at least 3 months prior to assessment, direct relationship with the dependent patient, provider of continuous care for at least 6 months, and previously included in the FCCP. 

If they presented any contraindication in their medical history or in the initial evaluation for performing moderate intensity exercise, they were excluded from the study. Other exclusion criteria were: inclusion in another program or activity that supports family caregivers, participation in regular physical activity (more than 3 days a week), having the help of one or more full-time formal caregivers to assist the dependent family member, or ceasing to be family caregivers (due to death, transfer, hospitalization of a dependent relative, etc.) during the time the study was carried out.

### 2.2. Sample Size Calculation

By including 68 individuals in the sample, we could obtain an 80% power to detect differences of two points when testing the equality of an SF-36 summary of physical health means, with a type I error of 5%. For this calculation, we assumed a standard deviation of approximately 7.46 and an attrition rate of approximately 15%. 

### 2.3. Randomization

Group assignment was performed by randomization after the selection of the participants. FFCs were assigned to the intervention (IG) or control (CG) groups. This randomization was performed by a simple random assignment method, for which a table/sequence of random numbers was generated using Microsoft Excel 2007^®^ software (version 12.0). 

### 2.4. Blinding

Due to the characteristics of the intervention, neither the FFCs nor the physiotherapist who delivered the PTE program could be blinded. However, the assessment performed by the research team was blinded. They did not know the allocated groups of the FFCs. The FCCP, which was common to both groups, was administered by healthcare professionals who also did not know which participants belonged to the IG. 

The statistician was blinded to the type of intervention assigned to each group, in addition to not being in contact with the participants.

### 2.5. Interventions

The participants in the intervention and control groups received the same conventional FCCP. The FCCP consisted of 4 sessions of 90 min for 1 month. The first three sessions comprised theoretical training, while the last session was theoretical and practical. They were taught by a nurse (1st and 2nd session), a social worker (3rd session), and a physiotherapist (4th session). Sessions were taught in small groups of 15 people. 

The intervention group received an additional 36 sessions of 1 h of the PTE program for 12 weeks (with a frequency of 3 sessions per week). The sessions were delivered to groups of 15–20 caregivers. The PTE program was always carried out under the same environmental conditions and by the same physiotherapist, who had extensive experience in delivering this type of program. The program was designed by a multidisciplinary team, taking into account the needs and characteristics of the caregivers, and according to the recommendations of the American College of Sports Medicine (ACSM) [33]. 

Aside from the FCCP, the control group received 6 extra phone calls over 3 months (2 calls per month) to reinforce the content taught in the sessions.

Further details of the FCCP and the intervention procedure are described in ref. [29].

### 2.6. Statistical Analysis

Statistical analyses were carried out using the R v3.2.3 statistical package (R Foundation for Statistical Computing, Vienna, Austria). Categorical variables were reported as means ± standard deviations, with median and minimum-maximum scores. Qualitative variables were reported as percentages. We calculated 95% confidence intervals (95% CI) for means and percentages. The effect size, corresponding to the comparison of IG with CG, was calculated using Cohen’s “d” statistic [34]. For its interpretation, we used the following criteria: very small d = 0–0.19; small: d = 0.20 to 0.49; medium: d = 0.50 to 0.79; large: d ≥ 80. To contrast the homogeneity of the initial characteristics in the individuals assigned to the two treatment groups, the Student’s *t* test was used for independent samples. The Chi-square test was used to test differences in percentages. At the same time, to compare the pain locations reported by the participants, we used odds ratio (OR) and their corresponding confidence intervals. Spearman correlation coefficients and their associated *p*-values were calculated to study the relationship between baseline and changed variables. The level of statistical significance was set at *p* < 0.05 for all hypothesis tests performed.

### 2.7. Outcome Variables

Data were collected following a standardized protocol. For the first aim, we analyzed the following variables:

Pain intensity: the analogue visual scale (VAS) was used with an unmarked 100 mm line [35].

Pain location: McGill’s pain maps were used to measure the location and extent of pain areas [36].

Lumbar disability: the Rolland–Morris questionnaire was used to evaluate the degree of physical disability derived from non-specific lower back pain. This is a self-reporting measure consisting of 24 questions. The maximum score is 24 points, where a higher score indicates a greater level of disability [37].

Neck Disability Index (NDI): this is a self-reported questionnaire that consists of 10 Likert items ranging from 0–5 (the total score is out of 50). A higher total score indicates a higher level of disability [38].

For the second and third objectives, we analyzed the following outcome variables: 

Quality of life: the SF-36 is a 36-item scale constructed to assess health status and quality of life. It generates an eight-level profile of functional health and well-being scores, as well as a psychometrically based mental and physical health summary. Each scale is transformed directly into a scale from 0 to 100. Higher scores indicate better overall quality of life [39]. We analyzed the mental summary component (MSC) and the physical summary component (PSC).

Anxiety: this was assessed using the Spanish version of the anxiety sub-scale of the Goldberg Anxiety and Depression Scale (GADS). This is a self-reported measure and consists of nine dichotomous (yes/no) items that measure anxiety symptoms that have been present in the last two weeks in adults. Scores range from 0 to 9 [40].

Depression: the Spanish version of the short-form 15-item version of the Geriatric Depression Scale (GDS) was used to evaluate FFCs’ depression. It is a self-reported questionnaire consisting of 15 dichotomous (yes/no) items. The total score is out of 15, and higher scores indicate worse depressive symptomatology [41]. 

Subjective Burden: this variable was assessed using the Zarit Carer Burden Interview (ZBI). It includes a total of 22 Likert-type questions ranging from 0 to 4 (total score: 0–88). Higher scores indicate greater caregiver burden [42].

Endurance: maximal oxygen uptake (VO_2_ max) was estimated using the 2 km walking test, following the guidelines of the Afisal-Inefc Battery [43].

Handgrip strength: this was assessed for both hands using a hand dynamometer (TKK 5401, Tokyo, Japan). The mean value of both hands (kg/m2) was considered the outcome [44].

For further information, please consult the full protocol described in ref. [29].

## 3. Results

The mean age of the caregivers being studied was 64.3 ± 7.6 years, with the youngest caregiver being 47 years old and the oldest being 76 years old. The highest percentage of participants (53.2%) were aged between 59 and 70 years. It should also be noted that 22.6% of the family caregivers were over 70 years old. They were mostly daughters (48.4%) or wives (37.1%) of the dependent relatives. With respect to marital status, 66.1% were married and 16.1% were single. Among the caregivers, 64.5% had a primary education, although 8.0% of the caregivers had no academic training whatsoever. Regarding occupation, most of the caregivers were not working at the time of the study, 40.3% were retired, and 27.4% had never worked. 

Regarding the characteristics of care provided, the FFCs had been giving care for an average of 5.3 years, for more than 12 h per day (in 72.6% of cases), and either without help or with the help of a family member (67.8%). According to the Pfeiffer questionnaire, the dependent relatives presented significant (41.9%) and moderate (23%) cognitive impairment. Care recipients presented an average score of 34.2 ± 21.8 on the Barthel Index, indicating severe dependency. According to this index, 30.6% and 48.4% of the dependent relatives had either total or severe dependence for the activities of daily living, respectively. 

No statistically significant differences (*p* > 0.05) were found among the sociodemographic variables of the caregivers between the two research groups.

### 3.1. Musculoskeletal Pain Characteristics

Table 1 describes the baseline musculoskeletal pain characteristics of the caregivers. The caregivers in the study described pain in the week prior to the initial evaluation, presenting a mean pain intensity of 62.0 ± 11.80 mm, according to the VAS scale. According to the clinical history, the first episode of musculoskeletal pain occurred on average 45.26 ± 26.85 months before the start of the study. As for the number of locations with pain, the mean was 2.58 ± 1.19 body areas with pain, the minimum being 1 and the maximum being 6, out of a maximum of 7 possible locations. No statistically significant differences were found between the groups (*p* > 0.05) among any of the variables mentioned in the preintervention evaluation.

Regarding the location of the pain, all caregivers reported having pain in some location in the week prior to the initial evaluation. Only 21% of the caregivers reported having pain in one location and the majority (80.0%) reported pain in two or more locations. Lower back pain and neck pain were the most frequently reported locations by the caregivers in the pain maps, with a prevalence of 69.4% and 56.7%, respectively (Table 2). 

Most of the caregivers in the sample population (71%) presented moderate pain intensity at the initial evaluation, according to the VAS scale. 

Table 3 shows the coexistence of pain in the body areas assessed for pain, highlighting that musculoskeletal comorbidity was prevalent. For example, among the caregivers who reported lower back pain, 60.5% also reported cervical pain, 27.9% reported pain in the shoulders, 18.6% reported pain in the hips, 37.2% reported pain in the knees, and 20.9% reported pain in the ankles and feet. The associations between pain in different body areas, measured by odds ratio, are shown in Table 4. The strongest association was between pain in the knee area and the presence of ankle and foot pain.

#### 3.1.1. Effects of the Intervention on Musculoskeletal Pain

In the pre-postintervention evaluation of the intensity of pain of the caregivers in the study (Table 5), there was an increase in the mean score (EVA scale) in the CG, although this difference was not statistically significant (*p* > 0.05). In contrast, there was a significant decrease in the intensity of pain (*p* < 0.001) in the IG. It is noteworthy that after the physical exercise program, all of the IG caregivers who had initially presented a pain intensity above 70 mm on the VAS scale (28.1%) managed to reduce their pain intensity below that level (severe pain). In the intergroup analysis, there was also a statistically significant difference in favor of the IG, with a large effect size (d = −2.66) (Table 5).

The lower back disability (evaluated through Roland–Morris questionnaire) of the CG caregivers increased significantly (*p* < 0.05) after the intervention, while it decreased significantly (*p* < 0.05) in the IG caregivers. The intergroup analysis also showed a statistically significant difference (*p* < 0.001) and a large effect size (d = −2.18) (Table 5).

Regarding the effects of the intervention on cervical disability, it should be noted that, as with lumbar disability, there was a significant increase (*p* < 0.05) in the CG caregivers. However, there was a significant decrease (*p* < 0.001) in the IG caregivers. Furthermore, 25% of the IG caregivers who initially presented some degree of cervical disability in the initial evaluation did not present any cervical disability in the post-intervention evaluation. In the intergroup comparison, the difference was statistically significant and the effect size was large (d = −2.49) in favor of the IG (Table 5).

#### 3.1.2. Effects of Intervention on Quality of Life

In terms of quality-of-life variables, MCS and PCS, there were statistically significant intragroup differences for the IG caregivers (*p* < 0.05 and *p* < 0.01, respectively). However, there was a very slight but non-statistically significant increase in the mean scores for the CG caregivers. There was not a statistically significant difference in the intergroup comparison for MCS; in contrast, a statistically significant difference was found for PCS (*p* < 0.001). The effect size was small for MCS (d = 0.36) and large for PCS (d = 1.17) (Table 6).

#### 3.1.3. Effects of Intervention on Psychological Well-Being

The pre-postintervention assessment of caregivers’ anxiety and depression showed statistically significant (*p* < 0.01) decreases (lower anxiety and depression) in the mean scores of IG caregivers. In contrast, there were slight but not statistically significant increases in the mean scores of CG caregivers. The intergroup differences were also statistically significant and had a large effect size in favor of IG for anxiety (d = −1.52) and depression (d = −1.37) (Table 6). 

After the physical exercise program, the IG caregivers achieved a decrease in the total subjective burden score (from 57.06 ± 13.45 to 48.46 ± 11.19), with a statistically significant difference (*p* < 0.001). In contrast to the CG caregivers, there was a 31.2% decrease in the number of IG caregivers who initially presented a “severe subjective burden” and a 25% increase in the number of IG caregivers who went from presenting to not presenting a subjective burden. The intergroup analysis (CG versus IG) showed a statistically significant difference (*p* < 0.001) and large effect size (d = −2.38) (Table 6).

#### 3.1.4. Effects of the Intervention on Physical Condition

In terms of the effects of the intervention on cardiorespiratory endurance, there was a significant (*p* < 0.05) decrease in VO_2_ max during the performance of the test (Afisal battery 2 km test) by CG caregivers. In contrast, there was a statistically significant (*p* < 0.05) increase in VO_2_ max for the IG caregivers. This difference was also found in the intergroup analysis (*p* < 0.001) with a large effect size (d = 2.44) (Table 6).

In relation to the maximum manual grip strength, there was a decrease in the manual grip strength of the CG caregivers after the intervention, while it increased in the IG caregivers, both differences being statistically significant (*p* < 0.05 and *p* < 0.01, respectively). In the intergroup comparison, a statistically significant difference was also found (*p* < 0.001) with a large effect size (d = 2.23) (Table 6).

### 3.2. Correlations between the Initial Situation and Changes in the Study Variables

Weak correlations (r < 0.30) were found between the participants’ age, the hours and months of care undertaken by the FFCs, and changes in the study variables. Likewise, the cognitive impairment and level of dependency in activities of daily living of the dependent relatives showed a weak correlation with changes in the study variables (r < 0.30). However, in relation to initial values of anxiety (Goldberg) and depression (Yesevage), caregivers who initially presented higher levels of anxiety and depression managed to achieve the most improvement in terms of pain intensity (r = 0.33 and r = 0.40, respectively) (Table 7).

The lower the caregivers’ initial physical health perception (PCS), the greater the decreases in lower back disability (r = −0.40) and cervical disability (r = −0.39). With respect to the initial values of mental health perception (MCS of the SF-36), the caregivers who initially presented lower MCS values were those who presented the greatest decrease in pain intensity (r = −0.43). The caregivers who initially presented a greater subjective burden were those who achieved the greatest improvement in pain intensity (r = 0.38). The greater the initial lower back disability, the greater the decreases in lower back disability (r = 0.77) and cervical disability (r = 0.41). Likewise, the greater the initial cervical disability, the greater the decreases in lumbar disability (r = 0.38) and cervical disability (r = 0.69). Cardiorespiratory endurance and handgrip strength had a weak or very weak correlation with changes in the study variables (r < 0.30) (Table 7).

## 4. Discussion

The main objective of the study was to determine and characterize musculoskeletal pain in FFCs, as well as to assess the effects of adding a therapeutic exercise program to an FCCP of the SACYL on the quality of life, physical condition, and psychological well-being of the FFCs. Additionally, we aimed to analyze correlations between the initial situation of the caregivers and changes in the outcome variables.

The results suggested that the implementation of a physical exercise program, related to primary care physiotherapy and in a group format, resulted in significant and clinically relevant improvements in psychological well-being, physical condition, and quality of life for the group of caregivers of dependent family members who were included in the caregiver care programs of two basic health areas.

The sociodemographic profile of the caregivers in the study could be summarized as a caregiver with an average age of 64.35 ± 7.56 years, who is related to the caregiver either as a spouse (37.1%) or a daughter (48.4%), who is retired (40.3%) or not working (27.4%), and with an academic level of primary education (64.5%). These data are consistent with the caregiver profile shown in most of the research carried out in this field [7,9,45]

The first objective was to determine and characterize the musculoskeletal pain of the caregivers. It is striking that all caregivers in the study presented pain. Pain may have been one of the main reasons why the caregivers were included in the caregiver care program of these centers. In addition, most of the patients had pain that had been developing for more than three months; therefore, and taking into account the latest IASP pain classification, we can state that the caregivers suffered from chronic pain [46]. Numerous studies [14,47,48,49] have referred to the noticeable presence of pain in family caregivers derived from the care of the dependent relative. However, few studies have carried out an evaluation and determination of this pain. Likewise, the methodology used for pain determination is highly heterogeneous, which makes it difficult to compare the results.

The caregivers in our study sample initially presented moderate pain intensity, very similar to that reported in studies by Prieto et al. [49] and Villarejo et al. [50]. In the study sample, all caregivers presented pain in some part of their body. In addition, the coexistence of musculoskeletal pain in several body regions was important. These data are very similar to those obtained by Darragh et al. [51] where 93.5% of the caregivers reported musculoskeletal pain in at least one part of their body and 82% reported pain in more than one location in the four weeks prior to the evaluation. As in the study by Darraght et al. [51], there was a higher prevalence of lower back pain and cervical pain in the caregivers in our study. The prevalence of musculoskeletal pain in the female caregivers in the sample population was higher than that reported by the general Spanish population in the European Health 2020 survey [4]. Several factors could be involved in the explanation of this high “musculoskeletal comorbidity.” First, several authors—Daraiseh et al. [52], Smith et al. [53], and Kindler et al. [54]—have shown that having pain in one body location increases the risk of developing pain in other locations, which could be due to the phenomenon known as “regional interdependence” [54]. This phenomenon could also arise from nociplastic pain mechanisms [55]. Physical factors could also be involved in this comorbidity, since there was a statistically significant correlation between the functional dependence of family members with pain intensity (r = −0.26) and the number of pain locations (r = −0.39). Psychosocial factors play a fundamental role in increased pain intensity and pain persistence [56]. In this sense, the caregivers in the sample population presented high levels of subjective burden, anxiety, and depression, which were correlated statistically and significantly with the intensity and number of pain locations; this could enable the persistence of pain. However, it should be noted that there is some debate regarding the temporal and causal relationship between pain and psychosocial factors [57].

Regarding lumbar disability, the initial results of the study were very similar to those reported by Prieto et al. [49], who also evaluated lumbar disability using the Roland–Morris questionnaire. We did not find any research in the scientific literature that evaluated cervical disability in family caregivers.

The FFCs in the sample population had low levels of disability, considering the high prevalence of lower back and neck pain that they presented. There is an open debate on this subject in the scientific literature, where some authors have found a correlation between pain and disability in the general population, while others have not [58]. This last case is known as the “pain–disability paradox”, which should be interpreted from the perspective of the “biopsychosocial” model [56]. In this model, lumbar and cervical disabilities are not exclusively determined by pain, but also by physical [57,59] and psychosocial aspects [60,61]. Thus, in our study, cervical and lumbar disability correlated in a statistically significant way with physical factors, such as the functional dependence of the dependent family member, the number of musculoskeletal pain locations, and the intensity of pain in the caregivers, and with psychosocial aspects, such as subjective burden, anxiety, and depression.

The physical exercise program managed to reduce both the intensity of pain and the lumbar and cervical disabilities of the family caregivers included in the study. We found only one other study that evaluated the effects of physical exercise on pain in caregivers [49]. In this study, there was a decrease in the intensity of pain in family caregivers in the IG (23%) and an increase in the intensity of pain of caregivers in the CG (14%), although these differences were not statistically significant. In our investigation, unlike that conducted by Prieto et al. [49], the differences were not only statistically significant among the IG caregivers, but there was also a large effect size and a decrease in the VAS scale >20 mm (23 mm), which is considered clinically relevant [62].

Regarding lower back disability, a reduction of 3.16 points was observed using the Roland–Morris questionnaire in the pre-postintervention evaluation of IG caregivers, which is considered a clinically relevant difference [63]. These results are in line with those found by Oesch et al. [64], who carried out a systematic review and meta-analysis of controlled clinical trials on the efficacy of exercise in relation to lower back disability, concluding that physical exercise has a significant positive effect on lower back disability. However, these authors were not able to conclude what types of exercises were the most effective in reducing lower back disability. The increase in lower back and cervical disabilities in the CG caregivers could be related to the increased perception of pain and deterioration in physical condition. Furthermore, according to the COST-B13 working group guidelines [65], education in caregiving (ergonomics and postural hygiene) and passive technique in general is insufficient to reduce lower back pain and disability. We believe that more research is needed in this regard. Another possible explanation of the large effect achieved in lower back and cervical disabilities concerns the supervision of the program performed. There is evidence that exercises supervised by health professionals are more effective, compared to simple instruction in the performance of the exercises [66,67]. 

In relation to the effects of adding a therapeutic exercise program to an FCCP, subjective burden, anxiety, and depression behave as a block against the rest of the variables that affect caregivers. This is due to the existence of a close and statistically significant correlation between them. Thus, in our study, levels of subjective burden, anxiety, and depression were negatively and statistically significantly correlated with the CSF and CSM scores of the SF-36. For instance, the perceived subjective burden and psychic functionality (anxiety and depression) of the caregivers were related to the deterioration of HRQOL, a fact also observed by Badia et al. [48]. Numerous studies have evaluated the effects of physical exercise programs on HRQOL in the general population [68]. Our results were also in agreement with the majority of studies carried out in adult and older women. 

With respect to correlations between the changes in pain and disability and the initial conditions of the caregivers, it should be noted that the changes were poorly correlated (weak correlation r < 30) with the conditions of care. In other words, there were no significant correlations between the hours of care, time spent caring for the dependent family member, and the pathology of the dependent family member and the pain and disability presented by the caregivers. On the other hand, there were significant correlations between pain and disability with individual aspects of the caregivers’ wellbeing (anxiety, subjective burden, and depression). No correlations were found between the initial physical conditions of the caregivers and improvements in pain intensity and disability after the program (r < 30). These results showed, once again, that musculoskeletal pain does not depend so much on biomechanical aspects as on psychosocial aspects [69,70]. After the physical exercise program, the caregivers who initially presented more anxiety and depression achieved significant improvements in pain intensity. Physical exercise programs, and not only pharmacological treatments, can be an important tool in treating anxiety and depression in caregivers. Regarding correlations between the initial quality of life of the caregivers and the effects of the program on disability and pain, caregivers who initially had a poorer quality of life achieved greater improvement in terms of pain intensity and disability. This result could be related to the close relationship between pain and disability and quality of life. It is clear that caregivers generate a direct impact on the public economic system. As future lines of research, it would be interesting to conduct new studies focused on education in pain neuroscience, as well as the cost-effectiveness of this type of program. Following the results of our study, FCCPs should not only focus on the physical well-being of caregivers, but also on psychosocial and individual aspects of caregivers. A more comprehensive approach would more effectively reduce these costs and improve the health and quality of life of caregivers. 

### Limitations

The main limitation of this study concerned the impossibility of performing a double blind study. To mitigate the fact that the results obtained were influenced by the caregivers knowing the group to which they belonged, masking was performed in the randomization, in the assessment of the caregivers, and in the data analysis; this was ensured by assigning a numerical code to each participant. Another factor to consider is that the study was only conducted with female caregivers. This means that the results obtained cannot be extrapolated to the male caregiver population; however, it was unfeasible for us to consider more healthcare areas. Lastly, follow-up was only performed up to week 20. A six-month follow-up would be desirable for future studies that apply this type of treatment.

## 5. Conclusions

The female family caregivers in the sample population presented musculoskeletal pain that had important repercussions on their quality of life. This musculoskeletal pain was influenced by both physical and psychosocial aspects. A therapeutic physical exercise program included in the caregiver care program improved the musculoskeletal pain of the family caregivers in a clinically relevant way. The caregivers who improved the most with the intervention were those who initially presented the most intense pain, those with the greatest level of disability, and those with the lowest quality of life.

## Figures and Tables

**Table 1:** Results of the initial evaluation of pain.

Outcome Measures	n	Mean ± and Standard Deviation	Median	Minimum–Maximum
Intensity of pain	62	62.01 ± 11.0	65.00	36.00–79.00
First episode of pain	62	45.26 ± 26.85	38	6.00–105
Number of locations of pain	62	2.58 ± 1.19	2.50	1.00–6.00

**Table 2:** Description of the sample population according to the number and location of pain.

Outcome Measures	n	%	95% CI
**Prevalence of the number of locations with pain**			
1	13	20.97	(11.66 to 33.18)
2	18	29.03	(18.20 to 41.95)
3	17	27.42	(16.85 to 40.23)
4	11	17.74	(9.20 to 29.53)
5	2	3.23	(0.39 to 11.17)
6	1	1.61	(0.04 to 8.66)
**Location of pain**			
Neck	37	59.68	(46.45 to 71.95)
Shoulders	24	38.71	(26.60 to 51.93)
Elbows–wrists–hands	15	24.19	(14.22 to 36.74)
Lumbar	43	69.35	(56.35 to 80.44)
Hips	12	19.35	(10.42 to 31.37)
Knees	19	30.65	(19.56 to 43.65)
Ankles and feet	10	16.13	(8.02 to 27.67)

**Table 3:** Coexistence of musculoskeletal pain (%) among different anatomical areas.

Location of Pain	Neck	Shoulders	Elbows–Wrists–Hands	Lumbar	Hips	Knees	Ankles and Feet
**Neck**	100	40.54	24.32	70.27	16.22	24.32	10.81
**Shoulders**	62.50	100.00	20.83	50.00	8.33	33.33	16.67
**Elbows–Wrists–Hands**	60.00	33.33	100.00	80.00	13.33	20.00	20.00
**Lumbar**	60.47	27.91	27.91	100.00	18.60	37.21	20.93
**Hips**	50.00	16.67	16.67	66.67	100.00	50.00	25.00
**Knees**	47.37	42.11	15.79	84.21	31.58	100.00	42.11

**Table 4:** Odds ratio (95% CI) of the associations between musculoskeletal pain in different anatomical areas.

	Neck	Shoulders	Elbows–Wrists–Hands	Lumbar	Hips	Knees	Ankles and Feet
**Neck**		1.21 (0.43 to 3.46)	1.02 (0.31 to 3.33)	1.11 (0.37 to 3.33)	0.61 (0.17 to 2.18)	0.48 (0.16 to 1.44)	0.38 (0.10 to 1.53)
**Shoulders**			0.74 (0.22 to 2.50)	0.23 (0.07 to 0.71)	0.25 (0.05 to 1.28)	1.23 (0.41 to 3.69)	1.07 (0.27 to 4.25)
**Elbows–wrists–hands**				2.06 (0.51 to 8.39)	0.57 (0.11 to 2.95)	0.48 (0.12 to 1.97)	1.43 (0.32 to 6.39)
**Lumbar**						2.85 (0.78 to 10.40)	2.05 (0.44 to 9.47)
**Hips**							
**Knees**							14.91 (2.76 to 80.51)
**Ankles and feet**							

**Table 5:** Results (mean ± standard deviation) of the effect of the intervention on the intensity of pain and lower back and cervical disability.

Dimension (Outcome Variables)	Group CG (n = 30) IG (n = 32)	Pre	Post	Intra Dif. (95% CI)	Inter Dif. (95% CI)	Effect Size
**Pain intensity** (EVA)	CG IG	60.70 ± 12.80 63.20 ± 10.70	63.01 ± 12.40 39.80 ± 12.80	−2.3 (−0.54 to 0.09) 23.4 (19.5 to 27.3) **	−25.7 (−30.6 to −20.81) ‡	−2.66
**Lumbar Disability** (Rolland–Morris)	CG IG	5.23 ± 2.91 5.56 ± 2.42	5.77 ± 2.67 2.40 ± 1.54	−0.54 (−1.01 to −0.56) * 3.16 (2.43 to 3.88) **	−3.70 (−4.54 to −2.83) ‡	−2.18
**Neck Disability** (NDI)	CG IG	13.67 ± 8.36 13.78 ± 7.04	14.68 ± 8.10 7.97 ± 5.33	−1.01 (−1.612 to −0.38) * 5.81 (4.55 to 7.07) **	−6.82 (−8.19 to −5.43) ‡	−2.49

Note: CG: control group; IG: intervention group; Pre: values before the intervention; Post: values after the intervention; Intra Dif: differences (intra-group) between Pre and Post at 95% confidence interval; Inter Dif: differences between the control and intervention groups at 95% confidence interval. * and ** indicate significant intragroup differences (*p* < 0.05 and *p* < 0.01, respectively); ‡ indicate significant intergroup differences (*p* < 0.01).

**Table 6:** Results (mean ± standard deviation) of the effect of the intervention on quality of life, psychological wellbeing, and physical condition.

Dimension (Outcome Variables)	Group CG (n = 30) IG (n = 32)	Pre	Post	Intra Dif. (95% CI)	Inter Dif. (95% CI)	Effect Size
**Mental Component** **Summary** (MCS)	CG IG	43.47 ±10.64 44.51 ± 8.27	43.26 ± 9.11 45.59 ± 7.22	0.20 (−1.29 to 1.70) −1.07 (−2.10 to −0.04) *	1.27 (−0.50 to 3.06)	0.36
**Physical Component** **Summary** (PCS)	CG IG	48.49 ± 6.52 47.57 ± 4.65	47.73 ± 5.67 50.20 ± 4.71	0.75 (−0.361 to 1.87) −2.63 (−3.62 to−1.63) **	3.38 (1.92 to 4.85) ‡	1.17
**Anxiety** (Golberg)	CG IG	4.30 ± 2.25 4.53 ± 2.01	4.47 ± 2.24 3.12 ± 2.13	−0.17 (−0.51 to 0.17) 1.40 (0.99 to 1.81) **	−1.57 (−2.09 to −1.05) ‡	−1.52
**Depression** (Yesavage)	CG IG	5.47 ± 2.69 5.91 ± 2.66	5.77 ± 2.82 4.41 ± 1.66	−0.30 (−0.61 to 0.01) 1.50 (0.90 to 2.09) **	−1.80 (−2.46 to −1.13) ‡	−1.37
**Subjective caregiver burden** (Zarit)	CG IG	54.93 ± 15.40 57.06 ± 13.45	56.67 ± 15.13 48.46 ± 11.19	−1.73 (−2.55 to 0.91) ** 8.59 (6.539 to10.64) **	−10.32 (−12.52 to −8.13) ‡	−2.38
**Endurance** (VO_2_ max) (mL·kg·min^−1^)	CG IG	23.29 ± 5.31 22.89 ± 3.98	22.57 ± 5.78 26.01 ± 4.40	0.72 (0.26 to 1.19) * −3.12 (−3.79 to −2.46) **	3.85 (3.05 to 4.65) ‡	2.44
**Handgrip strength** (kg·m^2^)	CG IG	41.43 ± 6.76 39.92 ± 7.42	40.74 ± 6.88 42.63 ± 7.67	0.69 (0.37 to 1.05) * −2.71(−3.42 to −2.01) **	3.43 (4.21 to 2.65) ‡	2.23

Note: CG: control group; IG: intervention group; Pre: values before the intervention; Post: values after the intervention; Intra Dif: differences (intra-group) between Pre and Post at 95% confidence interval; Inter Dif: differences between control and intervention groups at 95% confidence interval. * and ** indicate significant intragroup differences (*p* < 0.05 and *p* < 0.01, respectively); ‡ indicate significant intergroup differences (*p* < 0.01).

**Table 7:** Correlations between the initial situation of the caregivers and changes in the study variables.

Initial Conditions of the Variables	Pain Intensity	Lumbar Disability	Neck Disability
**Characteristics of care provided**			
**Age**	−0.01	−0.09	−0.24
**Hours of care**	0.01	0.25	0.26
**Months of care**	−0.12	−0.07	0.24
**Cognitive impairment DR** (Pfeiffer)	−0.1	0.32	0.31
**Dependency in activities of daily living DR** (Barthel Index)	−0.06	−0.21	−0.26
**Quality of life and mental health**			
**Anxiety** (Golberg)	0.33 *	0.3	0.26
**Depression** (Yesavage)	0.4 *	0.33	0.15
**Physical Component Summary** (PCS)	−0.22	−0.4 *	−0.39 *
**Mental Component** **Summary** (MCS)	−0.43 *	−0.13	−0.19
**Subjective caregiver burden** (Zarit)	0.38 *	0.29	0.31
**Physical Condition**			
**Endurance** (VO_2_ max) (mL·kg·min^−1^)	−0.06	0.12	−0.09
**Handgrip strength** (kg·m^2^)	−0.03	−0.11	0.07
**Disability**			
**Lumbar disability** (Roland–Morris)	0.18	0.77 **	0.41 *
**Cervical disability** (NDI)	0.16	0.38 *	0.69 **
**Pain intensity** (VAS)	0.33	0.33	0.11

Note: DR = dependent relative; * and ** indicate statistically significant correlations with a level of significance of *p* < 0.05 and *p* < 0.001, respectively.

## Data Availability

The datasets generated and/or analyzed during the current study are available from the corresponding author upon reasonable request.
